# Organizational boundaries of medical practice: the case of physician ownership of ancillary services

**DOI:** 10.1186/2191-1991-2-7

**Published:** 2012-04-05

**Authors:** John E Schneider, Robert L Ohsfeldt, Cara M Scheibling, Sarah A Jeffers

**Affiliations:** 1 Oxford Outcomes Ltd., Morristown, USA; 2 Texas A&M Health Sciences, Department of Health Management and Policy, College Station, USA; 3 Senior Director, Health Economics, Oxford Outcomes Ltd., 161 Madison Avenue Suite 205, Morristown, NJ 07960, USA

## Abstract

Physician ownership of in-office ancillary services (IOASs) has come under increasing scrutiny. Advocates of argue that IOASs allow physicians to supervise the quality and coordination of care. Critics have argued that IOASs create financial incentives for physicians to increase ancillary service volume. In this paper we develop a conceptual framework to evaluate the tradeoffs associated with physician ownership of IOASs. There is some evidence supporting the existence of scope and transaction economies in IOASs. Improvement in flow and continuity of care are likely to generate scope economies and improvements in quality monitoring and reductions in consumer transaction costs are likely to generate transaction economies. Other factors include the capture of upstream and downstream profits, but these incentives are likely to be small compared to scope and transaction economies. Policy debates on the merits of IOASs should include an explicit assessment of these tradeoffs.

This research was supported in part by funding from the American Association of Orthopaedic Surgeons (AAOS).

## Background

In recent years there has been considerable attention devoted to physician ownership of health-care related assets, with concerns raised over the effects of incentives associated with hospital ownership [1-7], ambulatory surgery center (ASC) ownership [8-10], and ancillary service ownership [11-14]. Ancillary ownership has been the focus of several recently published articles, and has been the focus of some state-led policy initiatives aimed at restricting physician ownership.^a ^Ancillary ownership generally includes ownership of free-standing ancillary services, such as imaging centers, as well as in-office ancillary services (IOASs), which typically includes standard imaging, advanced imaging, ultrasound, clinical laboratory, and outpatient therapy (e.g., occupational and physical therapy) [14].

In 1989 Congress adopted the Ethics in Patient Referrals Act, commonly referred to as Stark I, which went into effect on January 1, 1992. Stark I was amended in 1993, with the new amendments referred to as Stark II, going into effect on January 1, 1995. Stark II added some additional health services to the self-referral prohibition, extended the prohibitions to Medicaid, and clarified conditions for exceptions. The law was amended for a third time, with the new amendments referred to as Stark III, going into effect on December 4, 2007. Together, the current -Stark" laws prohibit physicians from referring Medicare patients to entities for certain -designated health services" if the physician, or an immediate family member of the physician, has a financial relationship with the entity. Financial relationships are broadly defined to include investment and ownership interests, as well as compensation arrangements between a physician, or his/her family member, and an entity [15-18].

The IOAS exception to Stark allows physicians to own in-office ancillary services. However, according to critics, the IOAS exception has created a loop-hole in the referral system and has contributed to recent growth in the utilization of in-office ancillary services [14,19-23]. Advocates of the IOAS exception say that the IOAS's purpose is to permit physicians to supervise the quality of care, to allow for better coordination among patients, physicians, and ancillary services, and to provide incentives for patients to adhere to recommended treatment plans. On the other hand, it has been argued that the exception creates financial incentives for physicians to increase the volume of services provided [14].

The purpose of this paper is to develop a conceptual framework through which to critically evaluate the tradeoffs associated with physician ownership of IOASs. Throughout the paper we use as an example the case of orthopaedic surgery practice ownership of imaging and physical therapy (PT). This case serves as a good example for two reasons. First, imaging and physical therapy are critical components of the orthopaedic continuum of care [24-28]. Second, orthopaedic surgeon ownership of imaging services has become a relatively common target of critics of physician ownership of ancillary services and medical facilities [13,29-36]. To explore the question of whether IOASs are -good or bad," the full set of tradeoffs should be considered. Setting aside the political aspects of the debate, the IOAS issue is an example of a common organizational problem faced by firms: whether to -make or buy" various stages of the production process.

### Boundaries of physician practice

**Figure 1:**
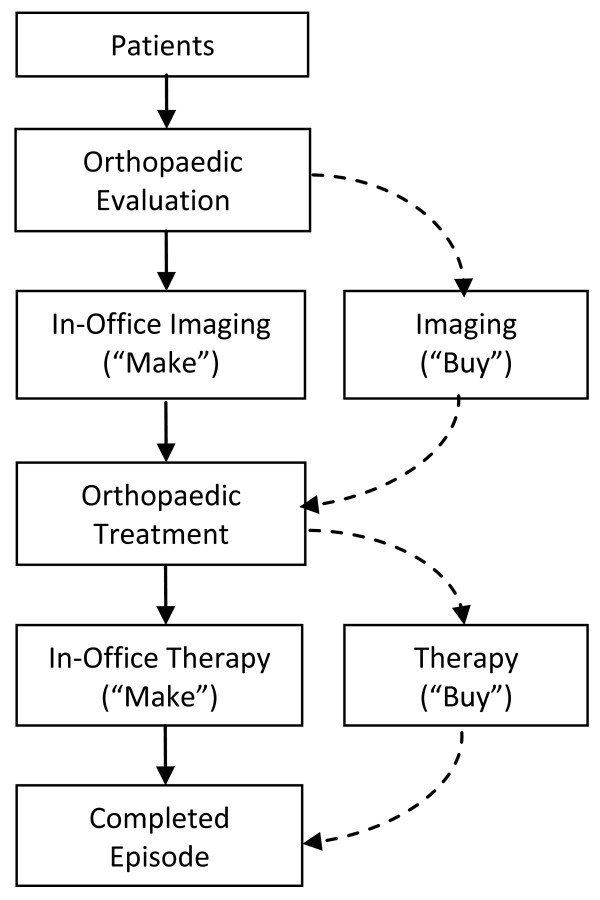
**Vertical Relationships in Orthopaedic Practices**.

The relationship between orthopaedic practices and ancillary services can be viewed as a -vertical relationship," where imaging can be viewed as an -upstream" service and physical therapy can be viewed as a -downstream" service relative to orthopaedic treatment (Figure 1). Vertical relationships can be organized through market-based contractual arrangements or through vertical integration, wherein the medical practice obtains imaging and therapy services via direct ownership.

The boundaries of firms-in this case whether medical practices should -make or buy" ancillary services-are a function of a variety of factors, mainly scope economies and transaction economies [37-43]. These factors can be thought of as -organic" drivers of the boundaries and scope of firms; the existence of these factors compels firms to adopt purposeful forms of organization designed to minimize operating costs and maximize comparative advantage. The following sections discuss these factors in more detail and in the context of orthopaedic medical practice and ancillary services.

### Economies of scope

Cases where the costs of conjoint production are lower than the costs of separate production are said to exhibit economies of scope, [44] and economies of scope are an important determinant of organizational boundaries [45,46]. Economies of scope are likely to be present when production relies on a common set of resources, such as technology, core competencies, and dual-use labor and capital inputs. Core competencies are particularly relevant to health care organizations, which rely disproportionately on highly trained human capital. Core competencies refer to firms' stock of knowledge assets (including tacit knowledge and know-how), skills, capabilities, learning processes, and resources. By diversifying and expanding into activities that are related to core competencies, firms are able to take better advantage of knowledge sharing, learning processes, and improved managerial efficiency [47-49].

Concentrating on scope economies and core competencies enhances the learning process by assuring that decision-making situations are repeated in sufficiently large numbers [50]. The role of learning depends on the ability of the firm to process information during the production process and apply the information appropriately. The learning process is critical to the formation and adaptation of organizational routines, which include rules of thumb, guidelines, templates, and protocols [51]. Specialized routines are the subcomponents of organizational -know how" and core competencies and are often sources of comparative advantage and production economies [52-54].

There are several aspects of orthopaedic IOASs that suggest the presence of economies of scope. Aiken et al. (2008) found that when orthopaedic surgeons work closely with PTs they were more likely to recommend non-surgical treatment options [55]. This finding was also observed during wartime, when military orthopaedic surgeons facing increasing caseloads were able to treat a larger volume of patients by triaging cases between surgical and non-surgical treatment pathways [56]. Similarly, there appear to be synergies associated with interdisciplinary teams conducting rounds at hospitals. In a study by Dutton et al. (2003), multidisciplinary hospital rounds for trauma patients included trauma center physicians, orthopaedic surgeons, physical therapists, and other members of the trauma care team [57]. The study found that the implementation of interdisciplinary teams reduced hospital lengths of stay by 36%.

The synergies observed in interdisciplinary teams are attributable in part to improved information flow. There are likely to be considerable benefits associated with improved flow of clinical information among members of the patient care team (orthopaedic surgeons, physical therapists, physician assistants, nurses, etc.) and between the care team and the patient. Often referred to as -corridor consultations," physicians and PTs can consult with one another with very little scheduling or planning [58].

One example of the benefits of better PT-physician communication is order clarification. There is some evidence that physicians typically provide non-specific and sometimes unclear referral diagnoses to PTs; according to one study, only 32% of physician referrals were judged to contain critical information regarding anatomy and pathology [59]. Orthopaedic surgeons and PTs practicing together have the potential to reduce these inefficiencies in knowledge transfer and face few impediments to doing so. In addition, physicians tend to underestimate pain and overestimate functioning [60], which suggests potential benefits from improved post-treatment communication between patients and physicians; in-office PTs offer a conduit for this type of information feedback.

### Transaction costs

There are two limitations to relying on scope economies alone to explain organizational boundaries. First, scope economies do not imply ownership; alternative forms of organization can be designed to capture economies of scope. Second, scope economies apply only to production technology and processes, and do not take into account demand-side factors. Transaction cost economics, however, has the advantage of addressing both supply and demand factors, and extends beyond scope and scale economies by focusing on the transaction as the unit of analysis [37,38,61,62]. The theoretical basis of transaction cost economics is that the boundaries of organizations are in part a function of the nature of the business transacted, where relatively complex transactions are more efficiently organized in settings that feature stronger administrative controls. Transaction complexity is a function of several factors; in health care settings, the most prominent forms of transaction complexity are uncertainty and imperfect information [63]. Uncertainty and imperfect information lead to increased production costs on the part of suppliers and increased search costs on the part of consumers.

All forms of governance exhibit strengths and weaknesses in organizing transactions. Uncomplicated transactions can be handled efficiently by organizational forms that lack strong controls, such as commodity markets and simple contracts. However, as transactions become more complex, they are most efficiently organized within forms of governance that offer stronger controls. In some cases, the most efficient means of organizing complex transactions is via full integration through ownership. The advantages of administrative controls, however, must be traded off with the increased bureaucratic costs associated with integration and, in the case of orthopaedic surgery practices owning IOASs, the possibility of added expenditures due to increased utilization.

In the market for medical care, consumer transaction costs are the costs incurred by the consumer to complete a transaction, including the time necessary to implement informed choice, such as evaluating, choosing and locating a care provider, as well as the time spent directly obtaining the services. Obtaining medical care requires non-trivial time input on the part of consumers, particular those over the age of 65. Based on our analysis of the 2009 American Time Use Survey (ATUS), Americans over the age of 65 devote approximately 108 hours per year in the primary activity of obtaining medical care services, including time spent obtaining, receiving, and purchasing services. Applying a median wage to this age group of $20 per hour,[64] this amounts to approximately $2,160 per person per year, or about 12% of total per capita medical care spending for the age group [65]. The effort required to obtain medical care is also reflected to some degree in -ease of access" measures. The Agency for Healthcare Research and Quality's report on National Health Disparities reported that 60-70% of the insured population -perceived difficulties or delays in obtaining care and problems getting care as soon as it is wanted." [66]

Although unmet need and pent-up demand are difficult to measure directly, there is some indication that these factors are important in IOAS demand and utilization. For example, in the case of PT, according to one study the proportion of patients with perceived need for PT but receiving no PT services increased during the two-year post-discharge period from 23% to 68%, suggesting substantial unmet need in physical therapy that increased with time from discharge [67]. In the case of imaging, the issue has more to do with the wide range of factors driving imaging decisions than unmet imaging need. For example, Carey and Garrett (1996) found that the use of CT and MRI for low back pain patients was associated primarily with patient characteristics, such as baseline functional status [68].

IOASs can be viewed to a certain extent as a response to these transaction costs. In the case of PT, these costs include finding a PT (or a set of potential or -feasible" PTs), determining the quality of the PT, determining the location of the practice and traveling to the facility. Consumer transaction costs are expected to be lower in the case of IOASs because patients have ready access to ancillary services and have the opportunity to economize on indentifying, vetting, locating and traveling to a provider [69]. In addition, there are several convenience-related benefits associated with IOASs, including easier scheduling, enhanced adherence to treatment plans [70,71], and -one-stop shopping."[58,72-75]

The latter benefit has been challenged in a recent study by Sunshine and Bhargavan (2010), who examined Medicare claims showing a visit to a specialist (an -index" visit) followed by an imaging claim associate with the same physician (i.e., a self-referred imaging service) [36]. The authors found that 74% of self-referred x-rays were conducted on the same day as the index visit, but only 15% of CTs and MRIs were conducted on the same day. The authors conclude that these data imply the absence of a -one-stop shopping" benefit to consumers, especially for CT and MRI. However, the study has two significant limitations. First, there is clearly a one-stop shopping benefit associated with some ancillary services, such as x-ray and ultrasound, which are relatively quick procedures unlikely to prolong an office visit. Second, the findings on CT and MRI most likely reflect the fact that advanced imaging requires more time, and prolonging a visit is not necessarily feasible for time-constrained patients. Moreover, these data do not reflect the likely convenience associated with on-site scheduling of CT and MRI scans and the reduced search costs associated with follow-up care (e.g., choosing providers, locating providers, and determining managed care network status).

Another important aspect of transaction economies is that medical care embodies -temporal specificities" in that diagnosis and treatment outcomes depend not only on *which *diagnostic test or treatment is performed, but also *when *the diagnosis or treatment is performed. Diagnosis and treatment timing is dependent on a variety of factors, including disease progression and symptoms, availability of providers, and patient adherence to treatment plans. For example, Gilbert et al. (2004) conducted a randomized controlled trial of patients with low back pain, and found that patients who received -early" imaging had better clinical outcomes than those who did not receive early imaging [27]. IOASs are a means of optimizing the timing of diagnostic testing (in the case of in-office imaging) and treatment (in the case of in-office PT).

### Other factors

Scope economies and transaction economies are the main drivers of vertical integration [43,76]. However, in some cases the motivation to vertically integrate may be driven simply by a desire to capture the profits of upstream and downstream firms. This view of vertical integration is the least prominent determinant of organizational boundaries, primarily because it lacks a -corporate coherence" component;[77] that is, were firms simply interested in acquiring profitable entities, the ownership of any profitable enterprise would suffice, and ownership of stocks, bonds and securitized assets are more convenient means of earning returns from profitable enterprises.

Nevertheless, critics of physician ownership of IOASs focus on this rationale in their depiction of vertical integration among medical practices and ancillary services. The crux of the argument is that asymmetrical and imperfect information allow some types of service professionals to -induce" demand for their services; that is, suppliers, -experts" in particular, have the ability to shift the demand curves of their customers [78,79]. In the medical care context, supplier-induced demand (SID) is defined as -the effect that doctors (or some other group of professionals), as providers of services, may have in creating more patient demand than there would be if they acted as perfect agents for their patients."[[80], p.333] Whereas several of the earlier studies of physician SID were hampered by study design and endogeneity problems (for a critique refer to Dranove and Wehner [81]), more recent studies controlling for endogeneity suggest that physicians are able to induce demand for their services to some extent, depending on the type of services and payment mechanisms [78,82-84].

There are two important limitations of relying on SID as a theoretical basis for understanding the effects of ownership. First, the better designed SID studies do not draw clear distinctions between differences in vertical relationships. Does full integration (via ownership) provide stronger inducement incentives than other forms of vertical relationships, such as joint ventures and long-term contracts? Afendulis and Kessler (2006) considered interventional cardiologists (i.e., those who perform diagnostic procedures and surgical treatment) as vertically integrated entities, and found that interventional cardiologists appear to be able to induce demand for angioplasty. However, their study did not distinguish among cardiologists who are full or part owners of cardiac catheterization facilities from those who utilize non-owned facilities via contract, such as hospital-owned facilities.

The second limitation of employing SID to critique integrated IOASs is that third-party payment mechanisms have evolved considerably in the past decade, and now typically contain a variety of administrative controls on utilization and payment. When coupled with the rapid diffusion of easily-accessible medical information and better-informed patients, administrative controls have placed limits on the intensity of SID [85-89]. In some managed care settings, ancillary service referrals require pre-authorization or pre-certification. This is especially true in the case of advanced imaging, such as CT and MRI. Thus, given that the literature generally indicates that the pre-certification process is effective in terms of reducing unnecessary services, [90-93] due to the -sentinel" effect of pre-certification review, it is unlikely that a substantial proportion of pre-certified or pre-authorized CTs or MRIs could be considered inappropriate.

It is also important to note that, empirically, it is difficult for researchers to accurately assess the clinical appropriateness of what may appear to be induced demand. Much of the research on ancillary service utilization is limited by inadequate controls for case mix severity and other demand factors. Consequently, these studies offer little evidence that higher utilization rates resulting from self-referral to ancillary services represent inappropriate or unnecessary care [94-96]. Thus, it is possible that increases in utilization in part reflect -pent-up" demand for services (i.e., services that would have been performed at higher rates prior to integration were there to have been sufficient capacity) rather than inappropriate care. For example, Restuccia et al. (1996) assessed whether the rate of inappropriate hospital admissions is higher in areas with higher rates of hospital admissions. Seventy small geographic areas were formed by grouping Massachusetts ZIP codes by similarity of hospital use [97].

Appropriateness of hospital admission was measured by applying an appropriateness protocol combined with physician judgment based on chart review. The authors found no relationship between hospital admission rates and inappropriate admission rates, calling into question the common assumption that areas with higher hospital use have more inappropriate use of hospital care.

Appropriateness also appears to be insensitive to financial incentives, even on the part of price-sensitive consumers [98,99]. Underscoring the fact that inappropriate care is a by-product of any medical care transaction, a study of the U.S. Veterans Health Administration (VA) hospital system found evidence of relatively high levels of inappropriate care in spite of the lack of financial incentives to physicians associated with VA hospital admissions [100]. Indeed, in their frequently cited study on IOAS ownership, Mitchell and Scott (1992) concede that -none of the studies to date...has been able to determine whether the increased utilization...represents inappropriate or unwarranted services."[34]

Finally, another important consideration is that IOAS integration implies the incurring of the operating costs of the acquired entity. In order to directly employ PTs, for example, orthopaedic surgeons must pay market salaries (which average approximately $72,900; see [101]). However, it is likely that salaried PTs will have somewhat lower productivity than self-employed PTs [12,102-104]. Thus, an orthopaedic surgery practice will have the same costs but earn less revenue than self-employed PT practices, the net result of which will be lower operating margins on PT services provided through the orthopaedic practice. Moreover, when compared to surgeons' own marginal revenue on resource-intensive procedures such as hip and knee replacement, it is unlikely that low operating margins on ancillary services provide sufficient financial incentives to invest in IOASs. For all services performed by orthopaedic surgeons, the 50^th ^percentile (median) billing rate per service is approximately $2,300, compared to a median billing rate per service of only $94 for PT (based on data reported in [105]).

## Discussion

The preceding section focused on two of the most commonly cited reasons for vertical integration-scope economies and transaction economies. Scope economies are likely to be an important factor in IOAS integration because of the potential to improve the flow and continuity of medical care. There is some evidence of scope economies in the case of physician integration into outpatient therapy. Transaction economies are also important to the integration decision, providing incentives for physicians to monitor quality (including the timing of ancillary services) and incentives for consumers to reduce the costs associated with obtaining medical care. There is some evidence of transaction economies in the case of physician integration into imaging and outpatient therapy. Other factors potentially influencing integration decisions include the capture of upstream and downstream profits, but these incentives are likely to be small compared to scope and transaction economies.

This leads to the pivotal policy question regarding tradeoffs: Are the economic benefits of integration worth the potential costs incurred through demand inducement and overutilization? To some extent policy makers have answered this question by passing a variety of laws restricting ownership, such as the Stark laws and the recently enacted federal Affordable Care Act (ACA), which places limits on physician ownership of acute-care hospitals. However, the language of these laws, and the content of the deliberations preceding the laws, generally lack explicit discussions of tradeoffs. Instead, the principal rationale has been simply that ownership leads to higher rates of demand inducement. In light of the preceding discussion, we argue that this view is too narrow, and has not allowed for the consideration of the benefits of integration.

The latter point is worth emphasizing given the rapid change in physician organizational arrangements over the past decade [106-110]. Imperfect agency is a central tenet of SID, but there are many factors that cause physicians to act as imperfect agents for their patients, including, for example: (1) information asymmetry between patient and physician; (2) financial incentives associated with third-party payment mechanisms; and (3) physician associations and business relationships (e.g., joint ventures and other collaborative arrangements; granting of admitting privileges; etc.) with medical groups, practice associations, and hospitals. Each one of these factors is enough to move physicians away from -perfect" agency on behalf of patients and toward some degree of imperfect agency [111-114]. The implication is that it is not simply ownership that influences clinical decision making and referral patterns, but a host of factors that are present in a variety of vertical relationships-not limited to vertical integration.

## Conclusions

The recent resurgence of controversy surrounding orthopaedic surgeon ownership of IOASs has been driven by a handful of studies, some of which have shown an association between orthopaedic surgeon ownership of IOAS and utilization of the owned services. In this paper we put forward a more balanced assessment of the tradeoffs associated with vertical integration in general and orthopaedic ownership of IOASs specifically.

Our main conclusions can be summarized as follows. First, any assessment of the effects of vertical integration should account for the full set of tradeoffs associated therewith, including the role of scope and transaction economies. One limitation of our paper is that we do not attempt to empirically measure these economies, and rely instead on anecdotal evidence in the literature. Future studies of physician integration into IOASs should measure these effects and present a more complete accounting of the tradeoffs.

Second, there is little upon which to base the assumption that SID works differently in ownership arrangements versus other forms of governance, such as joint ventures, long-term contracts, and other tightly coupled arrangements that may share some of the benefits of full integration. A limitation of the extant literature (and of this paper) is a lack of evidence comparing how SID intensity might vary by organizational arrangement. Until such studies are done, researchers should adopt a more conservative stance by allowing for possibility of differential SID effects across different organizational forms of medical care delivery. Similarly, although there have been some well-designed studies of SID in recent years, there is a need for continued methodological vigilance regarding SID studies, particularly with regard to the handling of endogeneity and the definition of ownership.

Third, even where these common empirical problems are overcome, the task of identifying the appropriateness of ancillary service use remains an important research challenge. Whether IOAS ownership is undesirable depends a great deal on whether induced services represent appropriate or inappropriate care. Given the lack of consistent findings on inappropriate care in other settings, there appears to be considerable room for further research on the extent to which added ancillary services are appropriate or inappropriate.

## Endnotes

^a^For example, refer to Maryland Court of Appeals decision in Potomac Valley Orthopaedic Associates, et al. v. Maryland State Board of Physicians et al. (January 2011).
